# Fluctuations of the elemental composition in the layers of mineral deposits formed on the elements of biogas engines

**DOI:** 10.1038/s41598-020-61212-x

**Published:** 2020-03-06

**Authors:** Ireneusz Stanuch, Maria Sozańska, Jolanta Biegańska, Jan Cebula, Jacek Nowak

**Affiliations:** 1 Municipal Plant of Residential Resources, Sosnowiec, Miejski Zakład Zasobów Lokalowych, Sosnowiec, Poland; 2 Department of Materials Research and Mechanics, The Silesian Technical University, Zakład Badań i Mechaniki Materiałów, Politechnika Śląska, Gliwice, Poland; 3 AGH University of Science and Technology, Cracow, AGH Akademia Górniczo-Hutnicza, Kraków, Poland; 4 Polish Academy of Sciences. The Szewalski Institute of Fluid-Flow Machinery, Fiszera 14 st., Gdańsk, 80-231 Poland; 5 Institute of Applied Geology, The Silesian Technical University, Instytut Geologii Stosowanej, Politechnika Śląska, Gliwice, Poland

**Keywords:** Mineralogy, Solid Earth sciences

## Abstract

The aim of the research was to compare the chemical composition and morphology of the surface of the backsheet growing on the metallic and surface substrate growing already on the prepared mineral substrate. Moreover the aim of this study was also to analyze the dispersion of chemical elements on the cross-sections of deposits formed on parts of landfill biogas-driven engines. The chemical composition of extreme layers of mineral deposits extracted from the engine piston and their cross-sections from four pistons and one head was examined by SEM-EDS. The bottom side showed a much smaller heterogeneity of the topography of the surface than the topside. The chemical composition of the deposit bottom side it primarily S (41%), Si (34%) and Al (17%). In the case of the top side, the dominated of Ca (52%) with a relatively high share of S (32%) and Si (14%). The presence of P and Mg it was also found, but only on the bottom side, and the share of Fe and Zn only on the top side. In the case of cross-sections, Ca, S and Si were the dominant elements. In general, there were higher Si participations in the zone of the bottom layer with a downward trend to the top sheet. The mass shares of S and Ca were lower in the zone of the bottom layer with the upward trend to the top sheet, also undergoing fluctuation.

## Introduction

In many countries, the acquisition of electricity and heat from renewable sources is an important component that complements conventional energy sources. The use of biogas among others for the production of electricity also fits in this trend^1^. Even small progress in extending the operation of gas engines and reducing the consumption of lubricating oils is of fundamental importance for renewable energy and environmental protection. Landfill gas used to supply power generators in cogeneration units^2^ contains various types of chemicals harmful to energy devices and lubricants. It shows relatively high variability of composition with respect to time of emission even within the same landfill. This is influenced by various factors ranging from the morphology of waste through their packing density, atmospheric conditions during storage and the time of storage. In addition to the basic components (methane, carbon dioxide), this gas also contains various substances, including those that have a destructive effect on the engine components. In addition to hydrogen sulphide in biogas, there are several hundred other compounds in trace amounts, among them organohalogen and organosilicon. They can cause the destruction of installations and devices for the energy use of biogas, raising the operating and investment costs for treatment by various methods^3–16^. Commonly occurring in sewage sludge organosilicon compounds - siloxanes play a special role. The presence of volatile methylsiloxanes (VMS) significantly reduces the efficiency of energy recovery from biogas. During biogas combustion, the silicon contained in VMS can combine with oxygen and various other elements found in the flue gas. At that time, deposits are formed on the engine components containing, among others, silicon compounds (e.g. silica, silicates) and other elements. They are visible in the form of a sediment with a smooth or rough structure in various shades of gray. They can create layers up to a few millimeters thick, which are difficult to remove. These mineral structures, which have abrasive properties, also include chemical compounds formed as a result of the influence of refining additives contained in engine oils^17^. The hard incrustations created in this way, e.g. on pistons, in the engine combustion chamber or valves, can destructively affect the functioning of the engine’s construction. These substances can lead to abrasive wear of moving parts of the engine and build –up of layers blocking heat conduction or significantly limiting lubrication. In practice, this can lead to equipment failures - very serious and expensive^18–24^.

Under normal operating conditions, a thin film of oil forms on the surface of the engine cylinder liner, which is intended to separate of sleeve from the piston ring. However, according to one theory^25^, when using aggressive gases containing, among others, silicon compounds, mineral structures that form during combustion can absorb the lubricant, reducing the amount necessary to ensure effective lubrication. This results in an insufficient amount of oil to produce the film and thus to secure the proper level of lubrication. Engine oil, which is an important structural element of the gas engine, changes its quality parameters during operation, which is a natural and unavoidable process. However, the lack of maintaining the appropriate thickness of the lubricating layer caused by excessive content of silicon compounds may lead to increased friction between cooperating surfaces. This may result in intensified changes in the properties of the oil leading to its accelerated degradation, which in turn may lead to faster wearing off engine components. Siloxanes are chemically transformed into various forms of silicon compounds that can be highly abrasive, leading to wearing between the moving surfaces of the engine. Particularly susceptible to this form of abrasion are the crankshaft and bearings, where hard particles can be trapped in their soft layers, which leads to the need for expensive grinding or even replacement of parts. These particles can cause scratches and even cuts on the surface of the sleeve and wearing on the piston rings^25^. Deposits can also cause changes in the geometry of the combustion chamber, causing an increase in the release of carbon monoxide and formaldehyde, possibly violation of the emission standards of pollutants into the air. In addition, detached fragments of deposits can block the cylinder liner and the growing layers inhibit the heat conduction and effective lubrication of the cooperating engine components. Deposits usually accumulate in the combustion chamber, on valves, valve seats, piston crowns and cylinder walls^26,27^. Taking into account the specific nature of the impact of deposits on equipment and the resulting possible economic implications, an important aspect of the impact on the system - engine and oil, seems to be the chemical composition conditioning the mineralogical structure generated during deposits growth. Information on the distribution of chemical elements during deposits’ build-up on cross-sections may indicate their ability to affect cooperating engine components at different engine operation periods. Fluctuations in the chemical composition on the deposit cross-sections may indicate the variability of the propellant gas components, as well as information on changes in the physic-chemical properties of the engine oil. The aim of the research was to determine the morphology of the surface and the elemental composition of deposit layers created during the combustion of aggressive biogas. The scope of the research included deposits created on front surfaces of four pistons, which were taken from the engine of a cogeneration unit specified as SC CHP.

Deposits formed in the combustion chamber were obtained from another cogeneration unit defined as GK CHP1. Both cogeneration units were operated at municipal waste landfills located in the southern and central Polish cities.

The research was carried out in order to compare the elemental composition of the layers of extreme deposits created in an engine fed with a biogas landfill. The intention was also to determine the differences and similarities in the chemical composition of deposits from various pistons of the same engine as well as a comparison of the results obtained with respect to deposits from the engine head from a different location. In the literature review carried out by the authors, no research papers describing the specificity of deposit layers were found. Deposit samples were collected from two engines of cogeneration aggregates of municipal waste landfills located in urban agglomerations in Poland. The morphological characteristics and elemental composition of deposit samples were analyzed using scanning electron microscopy (SEM) used in combination with energy dispersion spectroscopy (EDS).

## Objects and methodology of research

Deposit samples for research were taken from cogeneration units (SC CHP and GK CHP1) with average electric power below 1 MW powered of biogas from municipal waste landfills.

The following research works were carried out on the mechanically extracted material.

- Macroscopic examination of deposits - visual observation of the extreme surfaces and cross-sections of samples. Photographic documentation was performed.
- Research of deposits morphology and microstructure - using scanning electron microscopy (SEM).
- Research of the elemental composition of deposits - at points on extreme surfaces and cross-sections - by X-ray microanalysis (SEM-EDS) using an X-ray spectrometer with energy dispersion (EDS). The spectrometer was equipped with the THERMO NORAN SYSTEM SEVEN X-ray microanalysis system coupled with the HITACHI S-3400N scanning electron microscope. The voltage accelerating electrons −25 kV. The research was carried out at the Institute of Materials Science at the Faculty of Materials Science and Metallurgy of the Silesian University of Technology in Katowice. At the same time, due to the coupling of the X-ray spectrometer with the scanning electron microscope, it was possible to observe the morphology and microstructure of the deposits by analyzing images obtained from the secondary electron detector (SE) and backscattered electron detector (BSE). The Thermo Scientific software was used in the microscope to record images and X-ray microanalysis results. The Hitek program developed at the Department of Materials Science at the Faculty of Materials Science and Metallurgy of the Silesian University of Technology in Katowice was used to read the saved files, modify them and transform them into standard digital image formats. Studies on cross-sections of deposit samples were carried out after prior the execution of their microsection.

## Results

**Figure 1:**
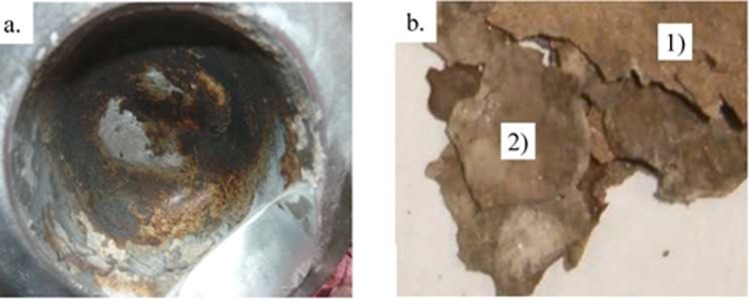
Sample 9T1-1/17 - the brittle incrustations of gray or gray-beige color with matte gloss, (**a**) piston head - place of sampling, (**b**) extracted deposits - top (from the combustion chamber) and bottom (from the piston head) side of the scrapings.

**Figure 2:**
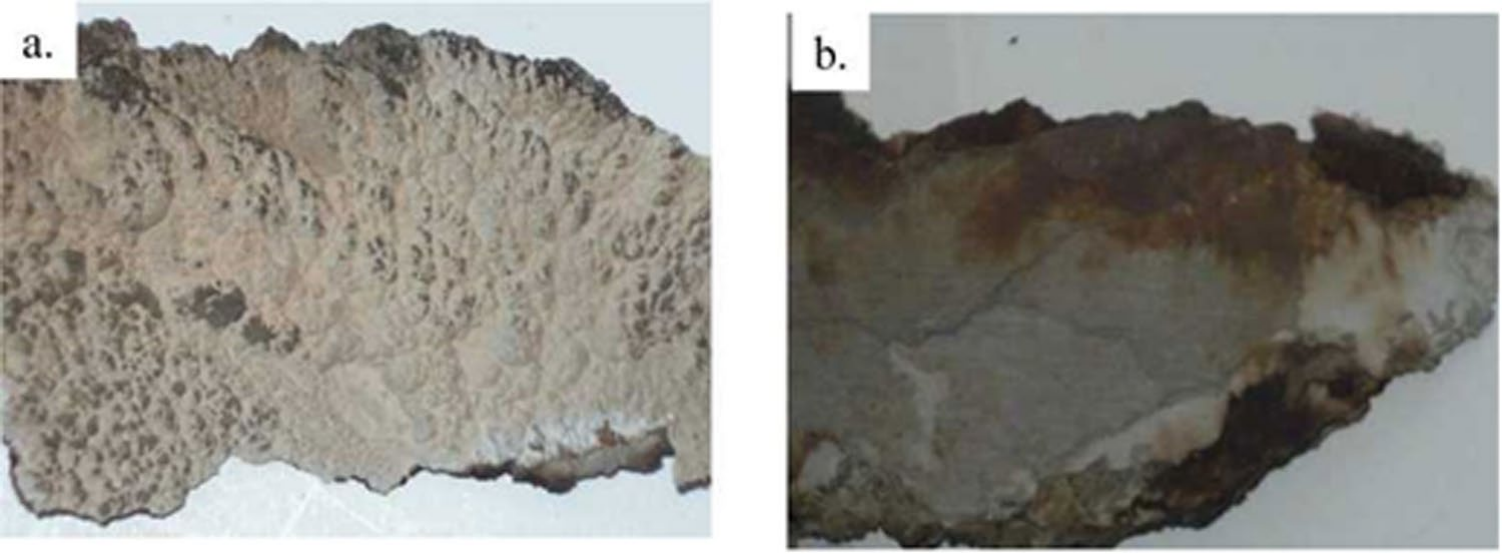
Sample 10T2-4/7, (**a**) top, rough side of the deposit - gray and beige incrustations. (**b**) the bottom, smooth side of the deposit - gray surface, locally beige and brown.

**Figure 3:**
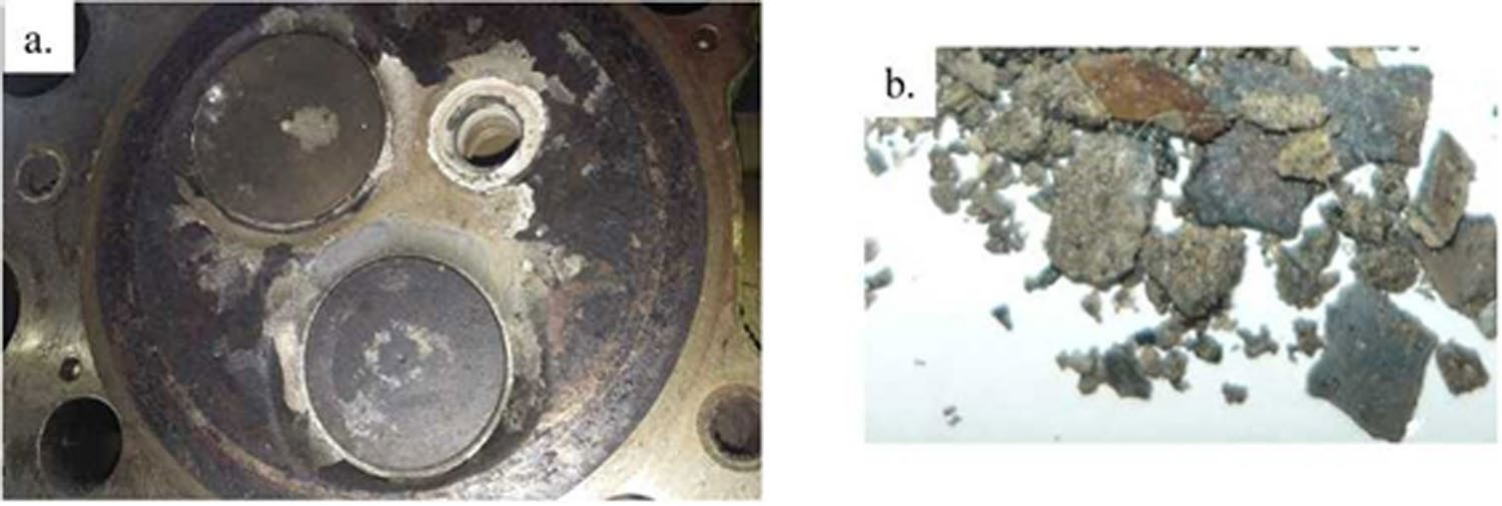
Sample 14K-5/9, (**a**) face of the head of engine with valves - place of sampling; visible deposit incrustations, (**b**) fragments of the deposit layer taken from the vicinity of the valve.

The samples were thin crusts of gray or gray-beige color, brittle and fragile with matte gloss. Examples of samples and their locations are shown in Figs. 1, 2, 3.

### Study of chemical composition of deposits using X-ray microanalysis (SEM- EDS) at points on extreme surfaces

**Figure 4:**
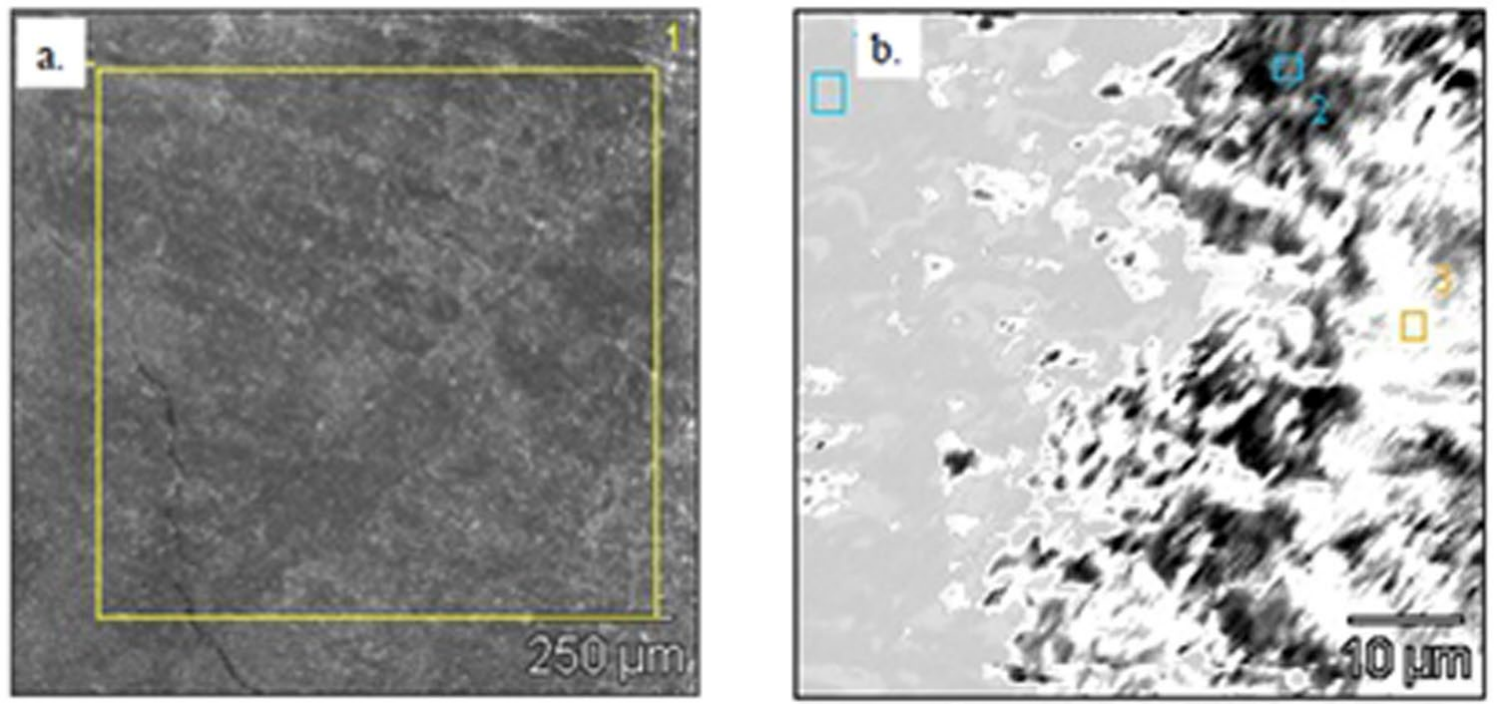
SEM EDS. Morphological characteristics in the points (Sp) of the underside of the deposit 9T1-1/17 observed at various magnifications; (**a**) Sp (1), X100, 250 pm; (**b**) Sp (2), X2000, 10 pm.

**Figure 5:**
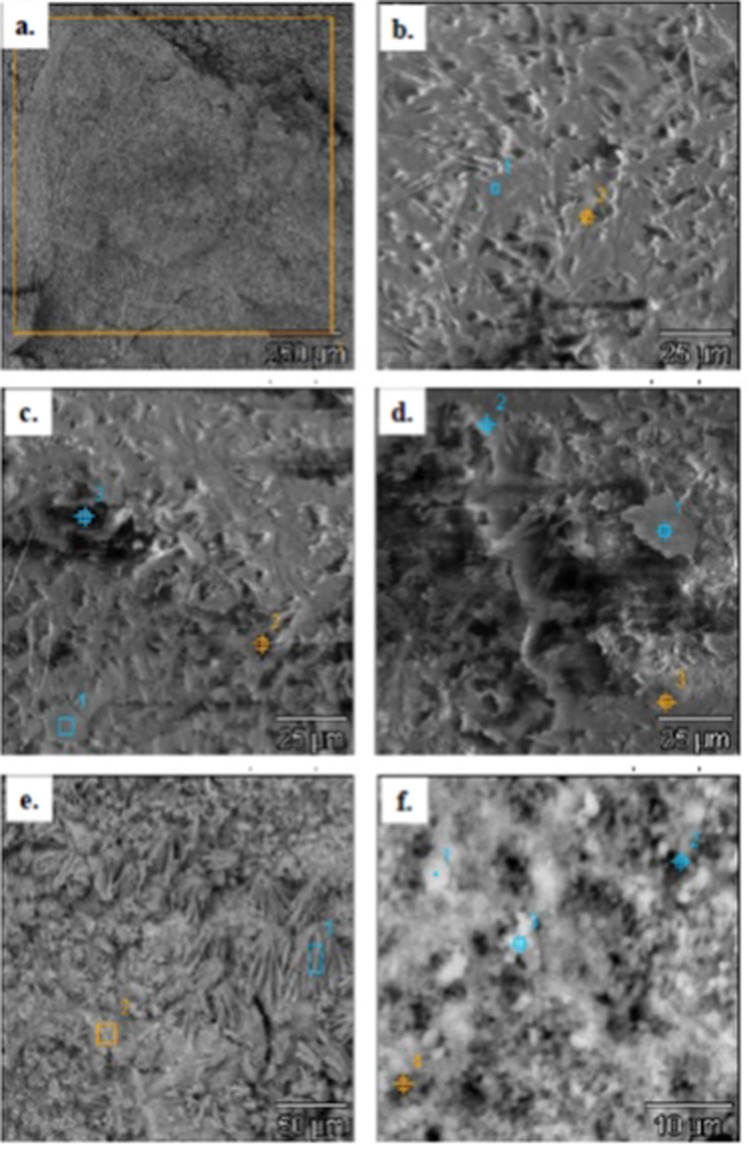
SEM EDS. Morphological characteristics at points (Chr) of the top side 9T1-1/17 observed at different magnifications; (**a**) Chr (1), X100, 250 pm; (**b**) Chr (2), X1000, 25 pm; (**c**) Chr (3), X1000, 25 pm; (**d**) Chr (4), X1000, 25 pm; (**e**) Chr (5), X1000, 50 pm; (**f**) Chr (6), X3000, 10 pm.

**Figure 6:**
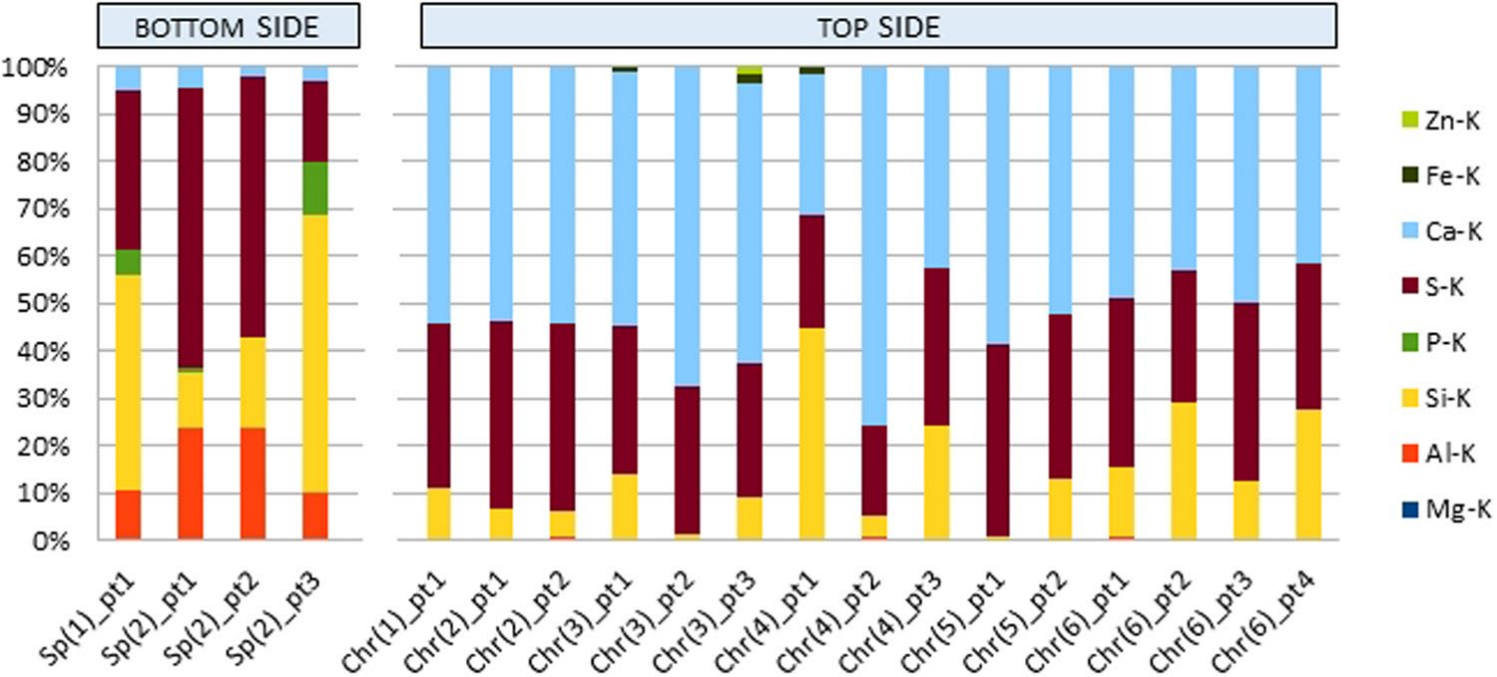
Quantitative composition of the chemical composition of extreme layers of the 9T1-1/17 sample - mass fraction [%]. SEM-EDS microanalysis.

The surface layers of the deposit sample 9T1-1/17 were subjected to surface structure studies and elemental composition. The layer adhering directly to the surface of the piston, was defined as a bottom side (smooth). The layer adjacent to the combustion chamber, i.e. lying on top of the deposit, (on which the deposit is built up), is designated as top (rough). In the photographs (Figs. 4a and 5f), the microstructures of the deposits external surfaces with the location of the study areas were presented. The results are presented in graphical form (Fig. 6) and in tabular form detail in Table 1.

**Table 1:** Chemical composition of extreme layers 9T1-1/17, weight share [%].

Bottom (Sp)/Top (Chr) side of the deposit		Sp (1)_ pt1	Sp (2)_ pt1	Sp (2)_ pt2	Sp (2)_ pt3	Chr (1)_ pt1	Chr (2)_ pt1	Chr (2)_ pt2	Chr (3)_ pt1	Chr (3)_ pt2	Chr (3)_ pt3	Chr (4)_ pt1	Chr (4)_ pt2	Chr (4)_ pt3	Chr (5)_ pt1	Chr (5)_ pt2	Chr (6)_ pt1	Chr (6)_ pt2	Chr (6)_ pt3	Chr (6)_ pt4
Element. Weight [%]	Mg-K	0,2																		
	Al-K	10,2	23,5	23,5	10	0,3	0,2	0,6	0,4	0,2	0,2	0,5	0,9	0,4	0	0,3	0,6	0,3	0,4	0,4
	Si-K	45,3	12,1	19,2	58,5	10,8	6,4	5,4	13,6	1,0	9,0	44,4	4,4	24	0,6	12,9	14,9	28,9	12,3	27,2
	P-K	5,8	0,9	—	11,5															
	S-K	33,5	58,8	55,1	17,0	34,5	39,4	39,4	31,1	31,3	28,3	23,9	19,0	32,9	40,7	34,4	35,6	27,5	37,3	31
	Ca-K	5,0	4,8	2,3	3,0	54,5	54,1	54,5	54,0	67,6	59,1	29,7	75,8	42,6	58,7	52,4	48,9	43,2	49,9	41,4
	Fe-K	—	—	—	—	—	—	—	0,9	—	1,7	1,5								
	Zn-K	—	—	—	—	—	—	—	—	—	1,8	—	—	—	—	—	—	—	—	—

#### The bottom side

In the investigation of the bottom layer of the sample (a square of 250 pm side), adhering directly to the surface of the piston, small irregularities of the analyzed area were found in relation to the other studies zones (Fig. 4a). At a magnification of X100, the differentiation in the size of depressions and bulges in comparison to the other side of the sample appears to be insignificant. The elemental composition (Table 1) indicates a weight share of primarily Si, over 45% and S above 33%. In addition, Ca and P were found above 5% and also slight amounts of Mg - 0.2%. The occurrence of aluminum with a mass fraction exceeding 10% as well as O and C in all scanned areas was also noted.

Three fields with optically different surface morphology were selected for analysis of the chemical composition at the points of this sample side (Fig. 4b). In the indicated locations the occurrence of mainly S was recorded, on average 43.6% and Si, almost 30%. On the other hand, the Ca ranked in all three locations ranging from over 2 to almost 5%. Disproportions of shares in Ca and S indicate that there is no calcium sulphate (Ca/S = 1.25). The increased S content is definitely higher than in gypsum or anhydrite. This may indicate that in contact with the metal elements of the S engine it does not bind to the Ca, or the binding occurs only to a very small extent. The presence of P was also noted in two points ranging from 0.9% to 1.3%.

#### The top side

The second, top side of the sample (rough) has a strongly developed surface compared to the previously tested. The scanned point was also a square with a side of 250 pm (Fig. 5a). In this case the elemental composition indicates the domination of Ca 54.5%, with a relatively large share of sulfur 34.5% indicating the possibility of anhydrite or gypsum. There was observed greater than on the side of the “smooth” the optical differentiation of the surface morphology, e.g. coniferous structures (Fig. 5b).

In the places selected for the analysis noted a significant differentiation in the mass share of Si from 0.6% to 44.4%, on average 14.4%. The smaller differentiation of the mass share was recorded in relation to S and Ca. S from 19% to 40.7%, on average 31%, and Ca from 29.7%, to 75.8%, on average 52.4%. On the other hand, the share of Al distributed in the range from 0.2%, to 0.9%, on average 0.4%. Fe was also found at the level of 0.2% to 1.7%, on average 1.4% and Zn with a share of 1.8.

### Summary of the results of microanalysis SEM-EDS of extreme deposits layers

Based on the investigations of the elemental composition of the 9T1-1/17 deposit sample in the extreme layers, there was a differences in surface morphology between the bottom layer adhering to the piston and the top surface in contact with the combustion chamber. The bottom layer showed a much lower heterogeneity of surface topography than the top which created various kinds of spatial forms. In most of the cases studied, irregular forms were observed but also the appearance of coniferous structures (Fig. 5b) and also of the lamellar type (Fig. 5d) were also observed. The existence of characteristic ordered conical structures with regular folds along the forming one was also registered (Fig. 5e). Such structures could suggest the impact of the wave of combustion gases from different sides. A strong differentiation of the elemental composition was observed between the bottom side and top side of the sample (Fig. 6). The strongest disproportions occurred in the Ca and Al ranges. In turn, P and Mg, only visible on the underside, and Fe and Zn on the top side. The circumstance of P disclosure only in the bottom side requires further investigation. The chemical composition of the this side indicated a strong participation primarily Si, S and Al. The proportions of Ca and S showed a definitely higher S content than in calcium sulphates (gypsum and anhydrite). This may point to the fact that S in contact with metal elements of the engine does not bind with Ca, or this bond occurs only to a very small extent. In the case of the face side, the elemental composition indicates the domination of Ca with a relatively high proportion of S indicating the possible of occurrence of anhydrite or gypsum.

The differentiation of the chemical composition of the extreme deposit layers and the presence of Al only on the underside can indicate that it comes from the alloy from which the engine piston was made. There is a relatively high probability that this element originates from a metallic layer of material, engine piston face (Al and Fe alloy). The presence of this metal can also be explained by the penetration into the motor oil of metallic abrasive, containing Al, also Fe, Cu, Cr, Sn or Pb. The typical source of Al in the oil are usually pistons, bearings, cylinder liner and engine blocks^28^. The compounds of this metal can also get from the outside in the form of mineral dusts together with the air sucked in. The source of Cu can be sliding elements such as sleeves and bearings as well as an oil cooler. Fe, in turn, goes into the oil most often from cylinders, crankshaft, timing, gears, bushings and bearings, and Pb can be released from the coatings to the bearings lapping, similar to Sn, which also penetrates from pistons and sleeves^28–30^. The appearance of Ca in the spectrum graph can be explained by the presence of its compounds in engine oil^31^. The content of Ca, P, Zn, Mg and B in the additives improves the oil’s functional properties, as rule, their quantity decreases during its use^29^. Ca is characterized by anti-wear and antioxidant properties, acts as a corrosion inhibitor and dispersant, in a similar way action magnesium. In addition to the dispersion properties, phosphor has an analogous attributes as an addition to oils operating at extreme pressures. Zn also has anti-wear properties, it is used as an antioxidant and corrosion inhibitor^30^. It can also, in a secondary sense, occur as an element brought in by the engine and more precisely by the bearings. However, S compounds can be both an oil component^32^ and a component of combusted biogas, e.g. H_2_S contained in a landfill gas.

Disproportions of Si share were found, this chemical element prevailed in the bottom layer comparatively to the top layer. Essentially, this element occurs in the form of silica and silicates, whose tendency to deposit depends on the substrates introduced into the engine as well as on the conditions prevailing in the combustion chamber^24^. Depending on the type of propellant gas, Si can penetrate into the engine oil together with dust brought with the air, which contains mineral Si compounds with relatively large particles. Organosilicon compounds are also present in biogas, e.g. volatile methylsiloxanes (VMS) and in antifoam agents introduced into the oil. The exact determination of the origin of Si in oil based on its analysis is difficult to determine.

Due to the discrepancy of the chemical composition of the outermost layers of the deposit, in order to determine the concentration of elements in the area between them, tests were carried out on cross-sections of samples.

### Study of chemical composition of deposits by X-ray microanalysis (SEM- EDS) at points on cross-sectional surfaces (EDS)

**Figure 7:**
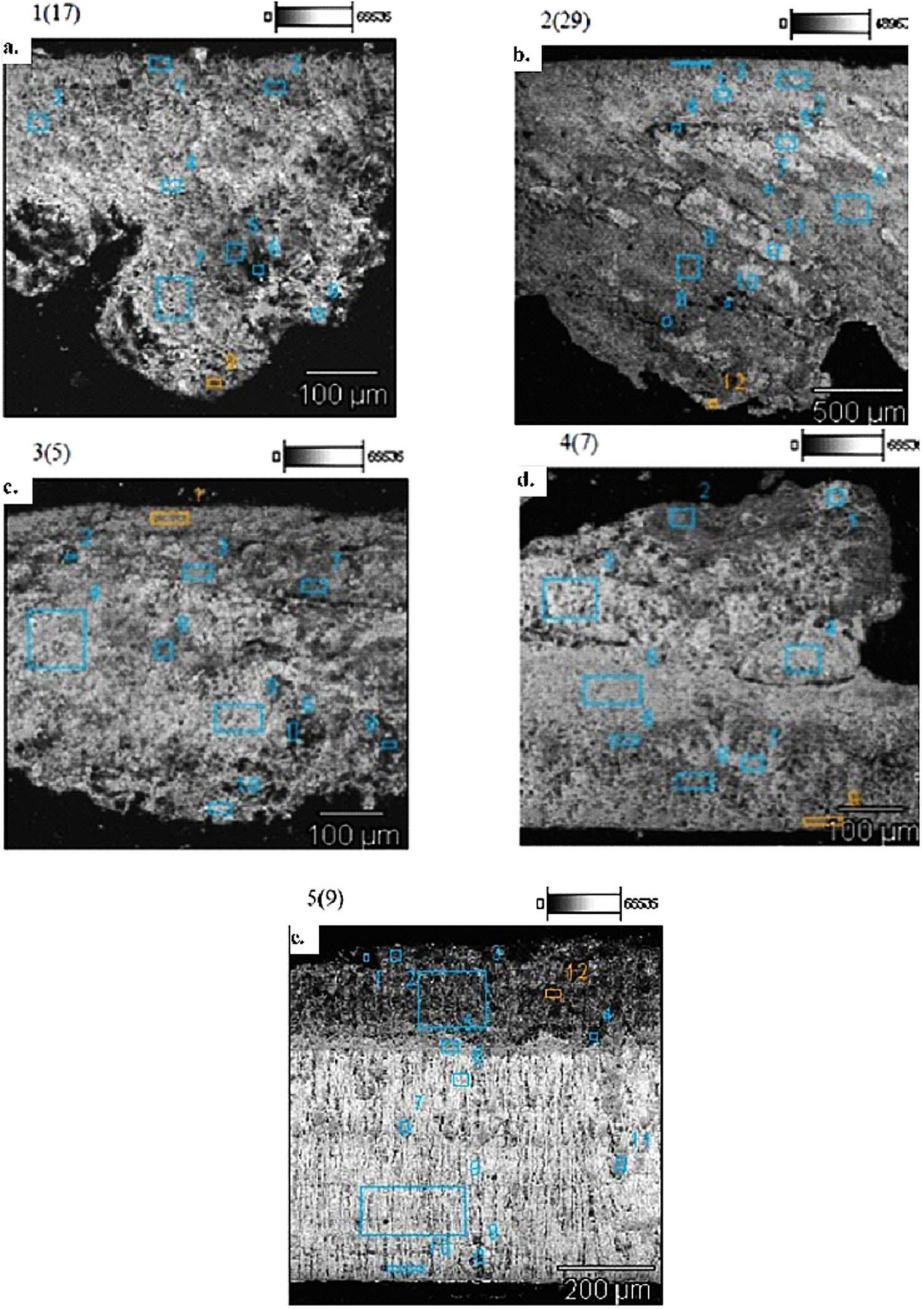
(**a**) Morphological characteristics of the cross–section 9T1-1/17, microanalysis points SEM EDS, magnification: X229, 100 gm. (**b**) Morphological characteristics of the cross-section 13T3-2/29, microanalysis points SEM EDS, magnification: X55, 500 gm. (**c**) Morphological characteristics of the cross–section 12T4-3/5, microanalysis points SEM EDS, magnification: X200, 100 gm. (**d**) Morphological characteristics of the cross-section 10T2-4/7, microanalysis points SEM EDS, magnification: X200, 100 gm. (**e**) Morphological characteristics of the cross-section 14K-5/9 microanalysis points SEM EDS, magnification: X160, 200 pm.

Research on the structure of layers and their chemical composition at selected points of cross-sections of deposit samples was subjected. The samples were specified as: 9T1-1/17, 13T3-2/29, 12T4-3/5, 10T2-4/7, 14K-5/9. The method of testing and the methodology of conduct are described in point 2 of this article. In the photographs (Fig. 7a–e) microstructures of the tested deposit cross-sections with the location of test points are presented. The results are summarized in Table 2.

**Table 2:** Elemental composition of micro-areas in deposit cross-sections - weight share [%].

No Fig.	Mikro-area	Chemical element. Weight[%].												
		Mg-K	Al-K	Si-K	S-K	K-K	Ca-K	Fe-K	Cu-K	Zn-K	Sn-L	Ti-K	Ni-K	Sb-L
Fig. 7a	9T1-1/17_pt1	—	1,2	65,9	16,4	—	16,5	—	—	—	—	—	—	—
	9T1-1/17 pt2	—	0,7	49,3	23,4	—	26,0	0,7	—	—	—	—	—	—
	9T1-1/17_pt3	—	0,3	26,2	32,4	—	41,1	—	—	—	—	—	—	—
	9T1-1/17_pt4	0,2	0,1	3,6	39,6	—	55,8	0,7	—	—	—	—	—	—
	9T1-1/17_pt5	0,4	0,6	17,6	56,8	1,0	20,1	1,2	—	—	2,2	—	—	—
	9T1-1/17_pt6	—	1,3	47,5	20,0	—	15,7	4,0	0,8	—	10,6	—	—	—
	9T1-1/17_pt7	0,3	0,4	3,9	42,0	—	52,6	0,8	—	—	—	—	—	—
	9T1-1/17_pt8	—	0,2	2,3	39,0	—	58,6	—	—	—	—	—	—	—
	9T1-1/17_pt9	—	0,4	5,1	38,1		55,6	0,7	—	—	—	—	—	—
Fig. 7b	13T3-2/29_pt1	2,3	—	40,1	34,9	0,4	22,0	0,3	—	—	—	—	—	—
	13T3-2/29_pt2	0,6	—	42,9	29,7	0,3	26,5	—	—	—	—	—	—	—
	13T3-2/29_pt3	0,4	—	14,5	35,6	—	48,5	0,4	—	0,6	—	—	—	—
	13T3-2/29_pt4	0,3	0,1	9,4	37,6	—	51,2	0,4	—	1,0	—	—	—	—
	13T3-2/29 pt5	0,5	—	5,8	39,3	—	54,1	—	—	0,3	—	—	—	—
	13T3-2/29_pt6	2,5	0,1	8,5	43,0	0,5	44,8	0,5	—	—	—	—	—	—
	13T3-2/29_pt7	3,3	—	9,4	42,5	0,6	40,8	1,5	—	—	1,8	—	—	—
	13T3-2/29_pt8	4,1	—	5,2	47,8	0,7	41,7	0,5	—	—	—	—	—	—
	13T3-2/29_pt9	7,6	—	1,3	65,1	2,1	23,9	—	—	—	—	—	—	—
	13T3-2/29_pt10	4,0	0,1	5,3	45,0	0,9	40,5	1,2	1,6	1,4	—	—	—	—
	13T3-2/29_pt11	1,0	0,2	3,3	40,9	—	53,9	0,6	—	—	—	—	—	—
	13T3-2/29_pt12	0,4	0,3	2,9	39,6		54,5	1,2	0,6	0,6	—		—	—
Fig. 7c	12T4-3/5_pt1	—	0,3	69,4	14,2		16,1	—	—		—		—	—
	12T4-3/5_pt2	0,3	2,7	38,0	24,6	—	25,4	3,0	0,8	—	5,2	—	—	—
	12T4-3/5_pt3	—	1,9	19,2	32,0	—	45,8	1,1	—	—	—	—	—	—
	12T4-3/5_pt4	—	0,7	5,8	36,3	—	53,2	4,0	—	—	—	—	—	—
	12T4-3/5 pt5	—	0,7	3,3	37,7	—	57,1	1,2	—	—	—	—	—	—
	12T4-3/5_pt6	0,2	2,8	21,5	30,1	—	37,3	5,3	1,2	—	1,6	—	—	—
	12T4-3/5_pt7	—	0,8	38,4	26,6	—	33,5	0,7	—	—		—	—	—
	12T4-3/5_pt8	0,3	2,8	37,8	24,9	—	25,7	2,7	0,6	—	5,3	—	—	—
	12T4-3/5_pt9	—	3,8	39,1	21,5	—	27,3	4,4	0,6	—	3,3	—	—	—
	12T4-3/5_pt10	—	0,4	1,4	38,3		58,6	1,3	—	—	—	—	—	—
Fig. 7d	10T2-4/7_pt1	—	0,3	3,4	38,4		57,3	0,6	—	—	—	—	—	—
	10T2-4/7_pt2	—	0,3	28,7	29,9	—	39,7	0,6	—	0,8	—	—	—	—
	10T2-4/7_pt3	—	0,2	5,4	37,0	—	56,9	0,5	—	—	—	—	—	—
	10T2-4/7_pt4	—	0,2	13,0	35,1	—	51,5	0,3	—	—	—	—	—	—
	10T2-4/7_pt5	—	0,2	26,5	29,6	—	43,0	—	—	0,7	—	—	—	—
	10T2-4/7 pt6	—	—	49,1	21,9	—	29,0	—	—	—	—	—	—	—
	10T2-4/7_pt7	0,2	0,2	27,7	29,5	—	41,6	0,8	—	—	—	—	—	—
	10T2-4/7_pt8	—	—	54,8	19,4	—	25,8	—	—	—	—	—	—	—
	10T2-4/7_pt9	—	—	72,4	13,9		13,7	—	—	—	—	—	—	—
Fig. 7e	14K-5/9_pt1	—	6,9	49,4	22,4	3,2	13,1	3,5	—	—	—	1,6	—	—
	14K-5/9_pt2	—	—	50,2	21,3	—	25,3	—	—	—	3,2	—	—	—
	14K-5/9_pt3	—	—	59,8	16,9	—	21,2	—	—	—	2,0	—	—	—
	14K-5/9_pt4	—	0,3	57,8	19,7	—	22,3	—	—	—	0,0	—	—	—
	14K-5/9_pt5	—	—	65,2	6,9	—	8,2	—	—	—	2,3	—	—	17,4
	14K-5/9_pt6	—	—	66,5	1,7	—	—	—	—	—	2,9	—	—	28,9
	14K-5/9_pt7	—	0,2	62,6	7,8	—	8,4	—	—	—	4,1	—	2,1	14,8
	14K-5/9 pt8	0,2		69,3	1,2						2,1			27,2
	14K-5/9_pt9	—	—	73,3	2,0	—		1,0	—	—	1,3	—	—	22,4
	14K-5/9_pt10	0,2	—	72,0	1,6	—		0,8	—	—	1,7	—	—	23,6
	14K-5/9_pt11	—	0,4	63,2	9,4	—	10,6	—	—	—	4,2	—	—	12,2
	14K-5/9_ptl2	—	—	66,1	15,6	—	17,1	—	—	—	1,2	—	—	—

#### The cross-section of the deposit 10T2-4/7

For the point analysis of the elemental composition of this cross-section, fields with optically differentiated surface texture were selected (Fig. 7a). At 229x magnification, fields with uneven gray levels in the analyzed area were distinguished. Attention was paid to poor stratification of the transverse surface and significant corrugation of the frontal. The elemental composition of the studied areas (Table 2) indicates a massive share of primarily Ca from 15.7 to 58.6%, an average of 38% and S, from 16.4 to 56.8%, an average of 34.2% and Si from 2.3 to 65.9%, on average 24.6%. In addition, the occurrence of Sn was found, ie 2.2 and 10% as well as Fe from 0.7 to 4%, on average 1.4% and Al from 0.1 to 1.3%, on average 0.6% and slight amounts of remaining elements in several places of the sample, i.e. Mg, K and Cu in the range of 0.1 to more than 1%. Oxygen and carbon were also found in almost all scanned areas. The scanned section points ran from the bottom side to the top deposit, as reflected by the increasing numbers of their markings (Fig. 7a). In the bottom layer of the adjoining to piston, the proportions of Si to Ca and S are much higher than in the folded top layer of the deposit. Taking into account the level of gray of the examined fields, it was observed that S definitely prevails in the darker places, and in these bright Ca and S in various proportions. Increasing the concentration of Sn (over 10%) and Fe (4%) and the occurrence of Cu (0.8%), can indicate the penetration into deposits of elements brought in by the alloys from which such engine components as bearings, pistons or sleeves are built. In turn, noting the presence of K, could indicate its origin from cooling water, to which this element is usually added as an anti-corrosive agent.

In the case of this particular sample, the deposit layers are not chemically uniform structures. Between the layers, one can see the boundary of their division, although it is not very clear. The inside of the layers contains areas of various sizes with different chemical compositions and levels of gray. Among themselves, the layers differ primarily with the content of the dominant elements, Si, Ca and S.

#### The cross-section of the deposit 13T3-2/29

Taking into account the morphological characteristics of this cross-section, attention was paid to the stratified diversity of the surface and the corrugation of the top deposit, as well as intra-stratified angular inclusions (Fig. 7b). The elemental composition of the studied micro-areas (Table 2) indicates a massive share of primarily Ca from 22 to 54.5%, average 41.9% and S from 22.7 to 65.1%, average 41.8% and Si from 1.3 to 42.9%, on average 12.4%. In all examined points magnesium from 0.3 to 7.6% was also visible, on average 2.3%. In addition, the occurrence of Sn - 1.8% and Fe from 0.3 to 1.5% was found, on average 0.7% and Al from 0.1 to 0.3%, on average 0.2%. The presence of K was also observed from 0.3 to 2.1%, on average 0.8% and Zn from 0.3 to 1.4%, on average 0.8% as well as Cu with a share of 0.6 and 1.6%. In addition, the occurrence of O and C in all scanned micro-areas was also noted. Taking into account the elements with the highest weight proportions, it was observed that in the bottom layer adjacent to the piston, the proportion of Si in relation to the surface layer is much higher. In turn, the Ca and S shares in the bottom layer are slightly smaller in relation to the surface layer, but the proportions between these elements in all points except one are similar. Taking into account the level of gray of the examined fields, no significant differences were observed in the chemical composition, also in clearly exposed angular inclusions of elongated shapes. In the case this particular sample, the deposit layers are not chemically uniform structures.

#### The cross-section of the deposit 12T4-3/5

Taking into account the morphological characteristics of this cross-section, attention was paid to the poor stratified diversity of the its surface, which consists substantially only of two layers that smoothly were overlapping, the bottom darker and the top one lighter. In addition, it was also observed to intra-stratified angular inclusions (Fig. 7c).

The elemental composition of the studied areas (Table 2) indicates on a decisive share primarily of Ca from 16.1 to 58.6%, on average 38% and S from 14.2 to 38.3%, an average of 28.6% and Si from 1,4 to 69.4%, average 27.4%. In all of the analyzed points, Al was also observed in the range from 0.3 to 3.8%, on average 1.7%. In addition the occurrence of Sn from 1.6 to 5.3%, on average 3.9% and Fe from 0.7 to 5.3%, on average 2.6% and Cu from 0.6 to 1.2%, on average 0.8% was found. Also the presence of Mg was observed, an average of 0.3% and there was also oxygen and carbon in all scanned areas.

In the case of elements with the highest weight proportions, it was observed that in the bottom layer of the cross section adjoining the piston, the proportion of Si in relation to the surface layer is much higher. In turn, the Ca and S shares in the bottom layer are smaller in relation to the surface layer, but the proportions between these elements at all points are similar. Taking into account the level of gray of the examined fields, no significant variation was observed in the elemental composition, except for the darkest points where the elevated degree of Fe and Al was recorded. Generally, for this particular sample, the deposit layers do not consist of chemically uniform structures that are clearly separated from each other.

#### The cross-section of the deposit 10T2-4/7

Considering the morphological features of this cross-section, attention was paid to the strong folding of the surface top layer of the deposit. In addition it was also observed strong stratification of this cross-section covering roughly three layers, with varying degrees of gray (Fig. 7d). The chemical composition of the studied areas (Table 2) indicates the dominant share of Ca from 13.7 to 57.3%, on average 39.8%, Si from 3.4 to 72.4%, average 31.2% and S from 13.9 to 38.7%, on average 28.3%. There was also observed the presence of Fe from 0.3 to 0.8%, on average 0.6% and Al from 0.2 to 0.3%, on average 0.2%. In addition, Zn 0.7 and 0.8% were found, as well as Mg 0.2%. Apart from the given composition, there was also the presence of O and C. Taking into account the elements with the highest shares, it was observed that in the bottom layer of the part adjacent to the piston, the proportion of Si in relation to the surface layer is much higher. In turn, the Ca and S shares in the bottom layer are significantly lower in relation to the surface layer, but the proportions between these elements at all points are similar. Generally, for this particular sample, the deposit layers do not constitute chemically uniform structures. Although they are clearly separated from each other, inside them various types of discontinuities and irregular forms can be seen.

#### The cross-section of the deposit 14K-5/9

In turn, considering the morphological features of this cross-section, attention was drawn to the strong stratified differentiation, which consists of three clearly separated layers with different degrees of gray (Fig. 7e). In the case of this particular sample, there is a clear optical separation of deposit layers. Schematically on the cross-section, the lighter layer can be defined as the bottom, the darker as the top one and the this medium gray level as the middle one. Although the layers are separated from each other and visually homogeneous, various types of small discontinuities and irregular forms can be seen inside them.

**Figure 8:**
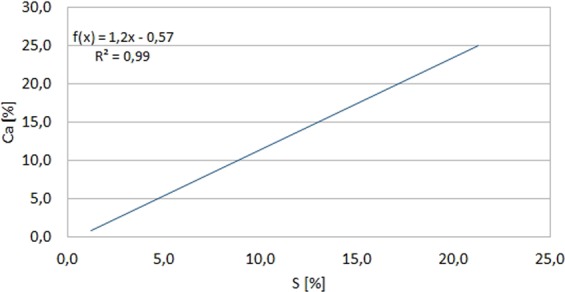
14K-5/9 deposit SEM EDS, linear regression Sb vs. S - weight share [%].

The chemical composition of the studied areas (Table 2) indicates the dominant weight share of Si from 49.4 to 73.3%, on average 63%, then Sb from 12.2 to 28.9%, on average 22.9%, Ca from 8.2 to 25.3%, on average 15.8% and S from 1.2 to 22.4%, on average 10.5%. Fe’s presence from 0.8 to 3.5% was also observed, on average 1.8% and Al from 0.2 to 6.9%, on average 2%. In addition, the presence of Sn was found from 1.2 to 4.2%, on average 2.3%, Mg 0.2% and also K - 3.2%, Ni - 2.1% and Ti - 1.6%. In addition, the occurrence of O and C in all scanned micro-areas was also noted. Taking into account the elements with the highest weight proportions, it was observed that in the bottom layer of the part adjacent to the piston, the proportion of Si in relation to the surface layer is much higher. In turn, the Ca and S shares in the bottom layer are lower in relation to the surface layer, but the proportions between these elements in all points (except the first) are similar. A very strong correlation of Ca and S shares was also found. Coefficient of linear regression R^2^ = 0,99 (Fig. 8).

**Figure 9:**
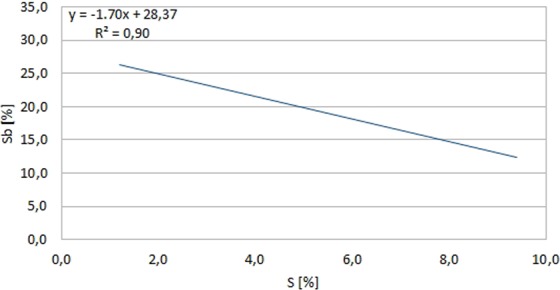
14K-5/9 deposit SEM EDS, linear regression Ca vs. S - weight share [%].

At the same time, it must be added that no Ca presence was observed at the four points of the bottom layer. On the other hand, the relatively high amounts of antimony in the middle and lower layers and its strong negative correlation with sulfur (R^2^ = 0,9) were observed (Fig. 9). This element is added to some engine oils as an EP (extreme pressure) exploitation formulation^33–35^ that ensures proper lubrication when transferring large of the stress. In addition, it can also be added to oils as an anti-wear agent and an oxidation inhibitor^36^. However, the source of its origin may also be the bearing alloys^37^. Attention was also paid to the presence of Ti and Ni in individual scanned points. Titanium can be used as an ingredient in the alloy of springs and valves^38^ and also as an additive to engine oils. Nickel is in turn included in the alloys used for valve seats of combustion engines^38^. Taking into account the gray level of the examined fields, it was observed that in the generally darker layer, there was a lower Si content relative to the clearer and intermediate, although this share in all layers was dominant. On the other hand, the shares of S and Ca, in contrast to the darker layer one, were the highest. There was also noted a clear chemical differentiation in the case of Sb shares occurring only in the bottom and middle layer and negative strong correlation of this element with sulfur. In this case, one can consider the option of changing the type of engine oil during engine operation.

#### Summary of the results of the SEM-EDS microanalysis of the cross-sections of deposits

On the basis of the conducted calculations of the surface area of deposits collected from engine components, defined as: 9T1-1/17, 13T3-2/29, 12T4-3/5, 10T2-4/7, 14K-5/9, it can be concluded that they are highly heterogenous in terms of chemical and structural. Microanalysis (SEM - EDS) on cross-sectional points of deposit samples showed the presence of morphologically distinct layers and areas with different levels of gray and varied chemical composition. Generally however, the surfaces of deposit cross-sections display weak stratification (except for 14K-5/9). Between the indistinct layers, you can see the boundaries of the division, although they are also not too exposed. The layers are not sharply separated, they penetrate each other to different depths. They contain inclusions of various sizes, usually from the matrix matter. In the case of clearly marked angular inclusions, one should consider the view of the possibility of detachment of already made earlier tiny fragments of deposits and their implementation into growing layers. The layers differ mainly in the content of the dominant elements: Si, S and Ca, and in sample 14K-5/9 also antimony, most likely, similarly to Ca from engine oil.

In samples cross-sections there are higher Si shares in the bottom layer zone with a downward trend to the top one, showing fluctuations of varying severity. In all the cross-sections examined, the S and Ca shares were lower in the bottom layer zone with the upward to the top one trend, showing also fluctuations. In the majority of micro-areas studied (with the exception of a few in samples 14K-5/9 and 13T3-2/29), it was found that the proportions of shares Ca and S were converge, despite their quantitative differences. In the case of Si, conversely, the shares of Ca and S fell at the points of growth of the share of this element.

There were no unambiguous relations found between the metal elements and Si except for the deposit sample 9T1-1/17, where there was a positive correlation with Al. In turn, in the sample 14K-5/9 there were strong positive correlation coefficients Ca and negative Sb in relation to S.

Considering the heterogeneity of cross-sections of samples in terms of morphology of the stratification and chemical composition, one can present the view that they result from variable engine operating conditions. Many factors can influence the characteristics and environment of machine operation, including composition of engine oil, intervals of its replacement and refilling, the stage of engine operation, the scope and frequency of repairs and the method of engine service, as well as the chemical composition of the fuel. Precise identification of the reasons for the lack of deposit isotropy may be a reason for further research.

## Conclusions

On the basis of the conducted research on surface morphology and cross-sections of deposit samples in points, one can draw the following conclusions:

1. Considering the dominant elements, one can argue that in the initial period of engine operation, silicon compounds are more easily deposited on metallic engine elements than calcium sulphates. In addition, the influence of silicon contained in the alloys of the part of the engine on the chemical composition of the bottom deposit layer can also be considered.
2. During engine operation, previously embedded structures of silicon compounds create better conditions for calcium sulphates deposition. Processes involving compounds silicon, sulfur and calcium run parallely, overlapping with different degrees of intensity, creating deposits with heterogeneous chemical composition.
3. In order to limit the growth of deposits with the dominant content of silicon compounds, consideration should be given to the use of properly selected engine oil refining additives suitable for various stages of engine operation. The presented view can be a prerequisite for further research.
4. The microanalysis of the chemical composition of deposits showed, among others the presence of phosphorus. This element may be present as a refining additive, but its participation only in the bottom layer requires recognition. It is advisable to clarify the content of phosphorus in the deposit, because compounds of this element can also penetrate into the landfill gas, (e.g. phosphine), demonstrating exceptional toxicity. Considering the above, the legitimacy to monitor the content of volatile phosphorus compounds in biogas should be considered.

## References

[1] Svensson, M., Hoyer, K. & Murphy, J. D. (Edited), IEA Bioenergy Task 37 - Country Reports Summary 2016. IEA Bioenergy (2017), http://task37.ieabioenergy.com/country-reports.html (2017).

[2] Gowrishankar, V., Angelides, C. & Druckenmiller, H. Combined Heat and Power Systems: Improving the Energy Efficiency of Our Manufacturing Plants, Buildings, and Other Facilities. NRDC, https://www.nrdc.org/sites/default/files/combined-heat-power-IP.pdf (2017).

[3] Allegue, L. B, Hinge J. & Allé, K. Biogas and bio-syngas upgrading. *Danish Technological Institute*, 5–97 (2012).

[4] Appels L, Baeyens J, Degrève J, Dewil R (2008). Principles and potential of the anaerobic digestion of wasteactivated sludge. Progress in Energy and Combustion Science.

[5] Bauer F, Hulteberg C, Persson T, Daniel T (2013). Biogas upgrading - Review of commercial technologies. Svenskt Gastekniskt Center AB, SGC Rapport.

[6] Beil, M. & Hoffstede, U. Guidelines for the implementation and operation of biogas upgrading systems. BIOGASMAX Integrated Project No 019795, http://biogasmax.co.uk/media/d3_5_iwes_biogasmax_v2_rev_nov2010068764100_1109_10022011.pdf (2015).

[7] Cebula, J. Biogas purification by sorption techniques. *ACEE Journal*, **2/2009**, 95–103, www.acee-journal.pl/cmd.php?cmd=download&id&id=82 (2016).

[8] Drosg, B. Process monitoring in biogas plants. IEA Bioenergy 2013, http://www.iea-biogas.net/files/daten-redaktion/download/Technical%20Brochures/Technical%20Brochure%20process_montoring.pdf (2017).

[9] Qie S (2015). Selection of appropriate biogas upgrading technology-are view of biogas cleaning, upgrading and utilisation. Renewable and Sustainable Energy Reviews.

[10] Ong, M. D., William, R. B., Kaffka, S. R. California Biomass Collaborative, University of. California, Davis. Draft Comparative Assessment of Technology Options for Biogas Clean-up. Contractor Report to the California Energy Commission (2014), https://biomass.ucdavis.edu/files/2015/10/Biogas-Cleanup-Report_FinalDraftv3_12Nov2014-2.pdf (2014).

[11] Petersson, A. & Wellinger, A. Biogas upgrading technologies - developments and innovations. IEA Bioenergy Task 37 - Energy from biogas and landfill gas 2009, https://www.infothek-biomasse.ch/images/175_2009_IEA_Biogas_upgrading_technologies.pdf (2017).

[12] Sevimoğlu O, Tansel B (2013). Composition and source identification of deposits forming in landfill gas (LFG) engines and effect of activated carbon treatment on deposit composition. Journal of environmental management.

[13] Surita, C. S. & Tansel, B. Preliminary investigation to characterize deposits forming during combustion of biogas from anaerobic digesters and landfill. *Renewable Energy* **80** (2015).

[14] Álvarez-Flórez, J. & Egusquiza, E. Analysis of damage caused by siloxanes in stationary reciprocating internal combustion engines operating with landfill gas. *Engineering Failure Analysis* **50** (2015)

[15] Guan B, Zhan R, Lin H, Huang Z (2015). Review of the state-of-the-art of exhaust particulate filter technology in internal combustion engines. Journal of Environmental Management.

[16] Al-Ghouti, M. A. & Al-Atoum, L. Virgin and recycled engine oil differentiation: A spectroscopic study. *Journal of Environmental Management* **90** (2009) 10.1016/j.jenvman.2007.08.018 18083292

[17] Sevimoğlu O, Tansel B (2013). Effect of persistent trace compounds in landfill gas on engine performance during energy recovery: A case study. Waste Management..

[18] Arnold, M. Reduction and monitoring of biogas trace compounds. VTT Tiedotteita - Research Notes 2496. VTT Technical Research Centre of Finland 2009, http://www.vtt.fi/inf/pdf/tiedotteet/2009/T2496.pdf (2017).

[19] Cars, A., Ozols, J. & VTtins, I. Getlini Project Gas Recovery and Power Production. *Eco Getlini*., http://www.getlini.lv/en/energija/Get_prezent_EN.pdf (2012).

[20] Guidance on gas treatment technologies for landfill gas engines. Environment Agency, http://www.gassim.co.uk/documents/LFTGN06%20Guidance%20for%20Gas%20Treatment%20v2%202010.pdf (2010).

[21] Soreanu G (2011). Approaches concerning siloxane removal from biogas - a review, Canadian Biosystems Engineering/Le Genie Des Biosyst. Au Canada..

[22] Stanuch, I. The effect of compounds of silicon on the formation of mineral deposits and on the degradation of the lubricating oil in engines fueled by biogas under real operating conditions (Doctoral dissertation 2017). Faculty of Energy and Environmental Engineering. Silesian University of Technology. Gliwice (2017).

[23] Micoli, L., Ausiello, A. & Turco, M. Treatment of Biogas for Feeding High Temperature Fuel Cells Springer International Publishing. (2016).

[24] Tower, S. & Tower, P. Guaranteed Removal of Siloxanes From Landfill and Digester. Applied Filter Technology, Snohomish, WA USA 2007, http://www.appliedfilter.com (2010).

[25] Carey V, Tellier K, Delafargue G, Squirrell D (2009). Product Development and Test Program For Aggressive Gas Engines. Mobil Industrial Lubricants.

[26] Ajhar M, Travesset M, Yuce S, Melin T (2010). Siloxane removal from landfill and digester gas - A technology overview. Bioresource Technology.

[27] Kaparaju Prasad, Rintala Jukka (2013). Generation of heat and power from biogas for stationary applications: boilers, gas engines and turbines, combined heat and power (CHP) plants and fuel cells. The Biogas Handbook.

[28] Malinowska M (2014). Engine oil pollution analysis used engine Cegielski-Sulzer 3AL25/30. Scientific Journal of Gdynia Maritime University..

[29] Przemysłowe środki smarne - Poradnik. Nadzór nad stanem maszyny i oleju. Total Polska **22**, 1–14 (2003).

[30] Signum Oil Analysis, Condition-Monitoring Fundamentals, http://www.signumoilanalysis.com/signum-english/Files/signum-oil-analysis-condition-monitoring-fundamentals-english-uk.pdf (2011).

[31] Ahmed, N. S. & Amal, M. Nassar Lubricating Oil Additives. Tribology - Lubricants and Lubrication, Dr Chang-Hung Kuo (Ed.). In Tech, http://www.intechopen.com/books/tribology-lubricants-and-lubrication/lubricating-oil-additives (2011).

[32] Litwiński, M. & Piec, P. Tribological propertis of engine oil in infrared spectroscopy aspect. Mechanika. Wydawnictwo Politechniki Krakowskiej. **14**, 127–33, https://suw.biblos.pk.edu.pl/downloadResource&mId=513807 (2012).

[33] Menezes, P. L. *et. al*. Tribology for Scientists and Engineers: From Basics to Advanced Concepts. *Springer Science*+*Business*, 295–333 (2013).

[34] Młynarczak A (2010). Preparaty eksploatacyjne stosowane w olejach smarowych. Scientific Journal of Gdynia Maritime University..

[35] Naveira-Suarez A (2010). Evolution of ZDDP-Derived Reaction Layer Morphology With Rubbing Time. Wiley Periodicals, Inc..

[36] Płaza, S., Margielewski, L. & Celichowski, G. Wstęp do tribologii i tribochemia. Wyd. Uniwersytetu Łódzkiego (2005).

[37] Przybyłowicz, K. Mataloznawstwo. WNT Warszawa (1999).

[38] Dobrzański, L. A. *Metaloznawstwo z podstawami nauki o materiałach*. Wydawnictwo Naukowo Techniczne (Warszawa 1996).

